# The Heating and Lighting of Hospital Wards

**Published:** 1909-03-27

**Authors:** 


### HOSPITAL ADMINISTRATION.

### CONSTRUCTION AND ECONOMICS.

### THE HEATING AND LIGHTING OF HOSPITAL WARDS.

#### VI.—RELATIVE ADVANTAGES AND COST OF METHODS OF LIGHTING.

As the writer understands the matter there are three points for consideration in connection with the lighting of hospital wards and hospitals generally: hygienic effect, convenience, and cost. The hygienic effect is, of course, by far the most important, but convenience has also an essential bearing, and unfortunately all hospitals have to consider cost. In the matter of convenience the question of lighting at night comes up. It is not sufficient only to give a good light in the evening, up to the time when the patients are settled for the night, but some light is wanted in the wards at night, and it must be of such a form as not to be obtrusive. In the matter of hygiene the electric light is far and away superior to anything else, inasmuch as it does not consume any of the oxygen in the atmosphere, and it does not deliver, in the smallest quantity, gaseous substances to the atmosphere as gas does. In the matter of convenience, taking the question of lighting by night into consideration, and the great developments that have taken place in gas fitting during the last ten or fifteen years, gas is somewhat superior to electricity. As explained in the previous article gas can be arranged to turn on from any point that is convenient, and can be turned down at night, whereas the electric light cannot except by special apparatus. In hospitals where the electric light is employed lamps are sometimes arranged in groups, each lamp taking its full current during full lighting time, but only sufficient to produce a red light at night. This requires special switching apparatus, which may get out of order. Special electric lamps are fitted with two filaments, one long, giving the full light when a good light is wanted and the other very short, giving a small light, similar to that given by a gas burner turned low at night. This arrangement has worked satisfactorily, but there is always a certain liability for it to get out of order, the space available for the necessary connections being small. Gas, whether burned in a mantle or in an open atmospheric burner, can be turned down as much as required, and the question of providing a smaller light at night is easily arranged. It should be noted, however, that with turned down gas burners, either open or with mantles, combustion is not so perfect, and there will be a much larger delivery into the room of unburned gas. As explained, gas is very much the cheapest form of illumination for any hospital that is within reach of a gasworks.

The new electric lamps take only about one-third the current taken by the old form of carbon filament lamps, but the price of the lamps themselves is high, their life has hardly been fully determined yet, and it is not always possible to realise the full benefit of the smaller current. The metallic filament lamps are not made in small candle-powers for the ordinary electrical pressures used in towns, and therefore more light and more current have to be employed in those towns where the service is by continuous current. In towns where alternating current is employed the difficulty is got over, and lamps down to 16 candle-power can be employed by the use of transformers. The transformers absorb a little of the saving which the new filament gives, but leave a substantial economy. The Nernst lamp, which had been introduced into some hospitals before the advent of the metallic filament lamp, and is an adaptation of the Welsbach mantle principle to electric light, will probably be extinguished by the metallic filament lamp. It has the disadvantage of being very complicated as compared with the ordinary incandescent lamp; it requires from half a minute upwards to light up, and its economy is not so great as that of the latest of the metallic filament lamps.

For country hospitals, sanatoria, etc., out of the reach of either gas works or electricity generating stations, probably the most economical method of lighting is by the new form of air gas, air with 1½ per cent. of petrol vapour. The cost of lighting by air gas is about one-third that of ordinary illuminating gas as sold in towns. It is made to furnish lights from 25 candle-power upwards, and it is used very much in the same way as ordinary gas is, with specially designed mantles. It has the disadvantage that except in one form of apparatus a machine is required to generate the gas, and some form of motor worked by water, oil, or other source of power is required to drive it. Next to air-gas the most economical is the method of lighting by delivering petroleum under pressure to mantles, in specially arranged lamps. The petroleum is carried in small pipes from a reservoir, where an air pressure is produced by a hand pump, to the burners, and is there vapourised by the heat of the burner itself, combustion and light taking place in the usual way.

The other method available for country hospitals is by means of petroleum lamps, which are made in practically all forms, and give light from very small power up to 100 candle-power and over. Petroleum lamps have been very largely improved of late years, particularly in the matter of the smell from the presence of the petroleum. The central draught lamps, in which the air passes up through the centre of the burner, and through the centre of the wick, and which some of us may remember as being the principle of the old "Moderator" lamp of days gone by, and of the Argand burner, burn very well indeed, and are very economical. It is claimed by lamp makers that a light of 50 candle-power approximately can be obtained for an expenditure of ¼d. per hour, at the average price of petroleum. One caution should perhaps be given in conclusion. Very much depends upon the quality of the oil employed. In country hospitals or sanatoria it is very dear indeed to use cheap oil. The oil should be specially suited to the work it is to perform, and be absolutely pure.

